# A link between mitochondrial damage and the immune microenvironment of delayed onset muscle soreness

**DOI:** 10.1186/s12920-023-01621-9

**Published:** 2023-08-23

**Authors:** Zheng Li, Lina Peng, Lili Sun, Juncheng Si

**Affiliations:** College of Sport Human Sciences, Harbin Sport University, No. 1, Dacheng Road, Nangang District, 150008 Harbin, China

**Keywords:** Inflammatory factors1, Sports injury2, Inflammatory pain3, Mitochondrial4, Immune cells5

## Abstract

**Background:**

Delayed onset muscle soreness (DOMS) is a self-healing muscle pain disorder. Inflammatory pain is the main feature of DOMS. More and more researchers have realized that changes in mitochondrial morphology are related to pain. However, the role of mitochondria in the pathogenesis of DOMS and the abnormal immune microenvironment is still unknown.

**Methods:**

Mitochondria-related genes and gene expression data were obtained from MitoCarta3.0 and NCBI GEO databases. The network of mitochondrial function and the immune microenvironment of DOMS was constructed by computer algorithm. Subsequently, the skeletal muscle of DOMS rats was subjected to qPCR to verify the bioinformatics results. DOMS and non-DOMS histological samples were further studied by staining and transmission electron microscopy.

**Results:**

Bioinformatics results showed that expression of mitochondria-related genes was changed in DOMS. The results of qPCR showed that four hub genes (*AMPK*, *PGC1-α*, *SLC25A25*, and *ARMCX1*) were differentially expressed in DOMS. These hub genes are related to the degree of skeletal muscle immune cell infiltration, mitochondrial respiratory chain complex, DAMPs, the TCA cycle, and mitochondrial metabolism. Bayesian network inference showed that *IL-6* and *PGC1-α* may be the main regulatory genes of mitochondrial damage in DOMS. Transmission electron microscopy revealed swelling of skeletal muscle mitochondria and disorganization of myofilaments.

**Conclusions:**

Our study found that skeletal muscle mitochondrial damage is one of the causes of inflammatory factor accumulation in DOMS. According to the screened-out hub genes, this study provides a reference for follow-up clinical application.

**Supplementary Information:**

The online version contains supplementary material available at 10.1186/s12920-023-01621-9.

## Introduction

Delayed onset muscle soreness (DOMS) is a cascade phenomenon that causes skeletal muscle pain and decreased muscle strength. In addition, in a significant proportion of patients it is accompanied by exercise fatigue, exercise haematuria, and other complications; serious cases will cause rhabdomyolysis [1]. These symptoms can affect the daily life of patients with DOMS [2]. Accumulating evidence shows that changes in immune cells are the main cause of myalgia [3]. Histologically, DOMS is characterized by infiltration of monocytes and natural killer (NK) cells [4]. The main characteristics of immune cells in the early stage of DOMS are circulating leukocyte counts and cell surface receptor expression [5]. However, the exact causes of the changes in immune cells associated with DOMS remain unknown.

In recent years, the research on exercise and immunity has attracted tremendous attention. In normal exercise, inflammation can promote muscle tissue repair and resist the invasion of pathogenic microorganisms [6]. However, exhaustive or unaccustomed exercise (particularly involving eccentric contractions) frequently results in temporary muscle damage, leading to DOMS [7]. Clinically, DOMS is characterized primarily by oxidative stress biomarkers and pro-inflammatory overexpression [7]. Tumour necrosis factor alpha (TNF-α) and interleukin-6 (IL-6) have been found to be the main biomarkers of DOMS. In particular, the inflammatory activity of skeletal muscle in DOMS increases linearly with increasing levels of TNF-α [8]. Although the mechanism by which DOMS creates an inflammatory milieu is unknown, this inflammatory response may be caused by the d isruption of cellular homeostasis.

Mitochondria are essential for maintaining cellular homeostasis [9]. Mitochondria are also a major source of oxidative stress products such as reactive oxygen species (ROS) [10]. Damaged mitochondria have been found to produce more ROS, which may explain the abnormally elevated biomarkers of oxidative stress in skeletal muscle in DOMS.

In this study, we combined the DOMS patient dataset from the NCBI Gene Expression Omnibus (GEO) with the MitoCarta3.0 database. We then screened for mitochondria-associated differentially expressed genes (DEGs). We used a computer-assisted algorithm to construct the DOMS mitochondrial gene and immune microenvironment network. In addition, we observed the mitochondrial phenotype and related genes in the skeletal muscle of DOMS and non-DOMS rats. On this basis, we used qPCR and Western blotting to verify the hub genes. This study explores the mitochondrial function of DOMS skeletal muscle and provides new insights into potential therapeutic targets.

## Materials and methods

### Preparation and analysis of rat triceps

All experiments were carried out on male Sprague–Dawley rats weighing 200–250 g (Harbin Medical University). The rats were randomly distributed into two groups: a non-DOMS group and a DOMS group after adaptive feeding for 1 week, with 12 rats in each group. The animals were kept in individual cages on a 12 h/12 h reverse light/dark cycle with free access to food and water. The rats were fed with NIH41 standard diet. All animal experiments were performed in accordance with the guidelines of the Animal Research Institute Committee of Harbin Sport University, Harbin, China.

The DOMS group started resting and exercising after the adaptation period. First, the DOMS group was familiarized with the treadmill, and the programme applied was 10 m/min on the first day and 12 m/min on the second day. After a 2-day adaptation period, the animals underwent a slightly modified running programme, at 25 m/min: speed run for 90 min (16° incline, 5 min/lap, and 2 min rest) [11].

The rats were anesthetized with pentobarbital (30–40 mg/kg) before decapitation. The bilateral triceps muscles of rats were rapidly cut into 4 mm-thick coronal tissue blocks. The left triceps were immediately flash-frozen in liquid nitrogen and stored at − 80 ℃. In each group, a cross-Sect. (1 × 1 × 4 mm) of the right triceps muscles of three rats was cut and fixed in 2.5% glutaraldehyde for follow-up experiments.

### Transmission electron microscopy

The muscle specimens were immersed in 2.5% glutaraldehyde for 2 h. The samples were shaved with a vibrating knife (Leica, Wetzlar, Germany) and immersed in 1% osmium tetroxide for 1 h at 4 °C. The samples were dehydrated stepwise with ethanol, embedded in epoxy, and sectioned at 70 nm using an ultramicrotome (RMC, USA). Finally, they were stained with uranyl acetate and lead citrate, and photographed using a transmission electron microscope (TEM; H-7650, Japan). Five serial sections were randomly selected for each skeletal muscle-embedded block.

### Western blot analysis

Proteins were extracted from cell or tissue samples for Western blot analysis using the RIPA buffer method. The samples were treated with RIPA buffer containing protease inhibitors, such as PMSF (Phenylmethylsulfonyl fluoride) or a cocktail thereof. After centrifugation, the supernatant was collected as the protein extract. Throughout the extraction procedure, the samples were kept on ice to minimize protein degradation and oxidation. The extraction process was carried out with gentle mechanical disruption and at a low temperature to preserve protein integrity and activity. Protein samples were boiled in 1 × sodium dodecyl sulfate (SDS) buffer to denature proteins and separated using gradient SDS-PAGE 10% gels [12]. The protein was transferred onto a polyvinylidene fluoride (PVDF) membrane using a standard wet transfer system with ice-bath, and the voltage was set to 100 V. Membranes were blocked with 5% skimmed milk for 1 h and then incubated with an antibody for 12 h at room temperature. Excess antibody was washed three times with Tris-buffered saline with Tween-20 (TBS)-T (50 mM Tris pH 8.0, 150 mM NaCl, 0.1% Tween 20). The following antibodies were used: AMPK (Beyotime, Beijing), PGC1-α (Beyotime, Beijing), and β-actin (Beyotime, Beijing). Peroxidase-conjugated secondary antibody (1 : 5000) was used as the secondary antibody (Bioss, Beijing). All antibodies were used at a 1 : 750 dilution in 5% non-fat dry milk. Band densitometry for western blots were quantitated using ImageJ software.

### Data acquisition and preprocessing

**Fig. 1:**
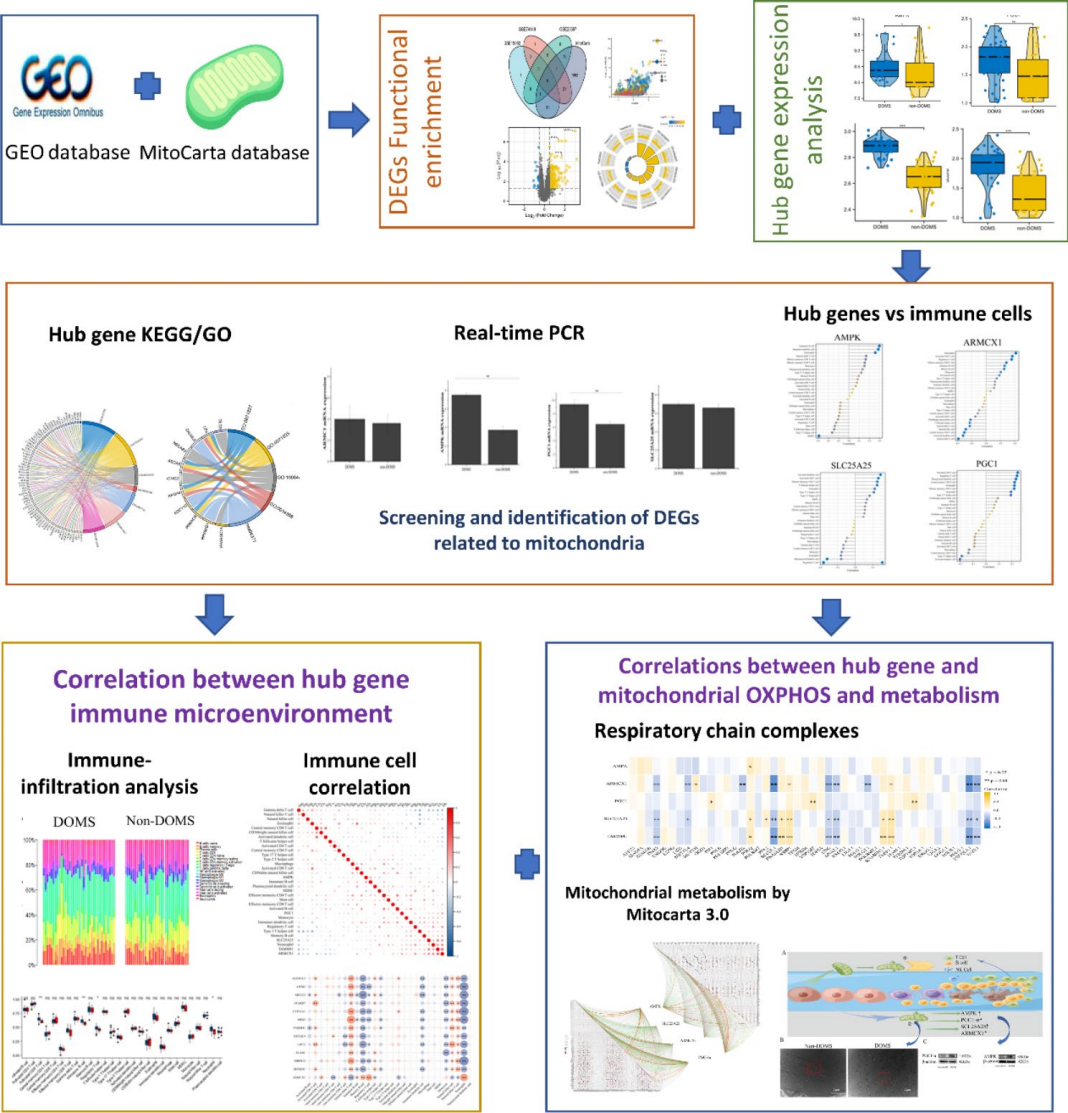
Flow chart of this study which focuses on the relationship between delayed onset muscle soreness (DOMS) mitochondrial dysfunction and the immune microenvironment

The flow chart of experimental data and bioinformatics analysis is shown in Fig. 1. The DOMS cohorts with publicly available data sets are from the GEO database, including GSE19062 (16 non-DOMS samples, 16 DOMS samples) and GSE74194 (10 non-DOMS samples, 10 DOMS samples). In addition, we utilized GSE23697 as a validation data set. GSE23697 is RNA-seq data containing 30 skeletal muscle samples from DOMS patients and 30 skeletal muscle samples from non-DOMS patients.

### Quantitative real-time PCR

RNA was isolated with an RNA extract kit (TransGen Biotech, Beijing). The extracted RNA was subjected to first-strand synthesis cDNA using a kit (TransGen Biotech, Beijing). The list of primers is compiled in **Supplementary Table 1**. GAPDH was used as an internal reference. Expression was calculated using the 2^−ΔΔCt^ method. We used BIOER ™ Thermal Cycler 9500 for qPCR analysis in this study. All PCR reactions were repeated in triplicate. Each well was loaded with a total of 20 µL containing 2 µL of cDNA, 2 × 0.5 µL of target primers, 7.2 µL of water, and 10 µL of SYBR Fast Master Mix. We performed hot-start PCR for 40 cycles, with each cycle consisting of denaturation for 5 s at 94 °C, annealing for 15 s at 58 °C, and elongation for 10 s at 72 °C. We used cyclophilin expression to normalize the mRNA expression.

### Identification of mitochondria-related DEGs and functional enrichment analysis

The R package ‘limma’ was used to screen for DEGs, setting a threshold of log2 | fold-change | ≥ 1 and a p-value < 0.05. Visualization of the overlapping DEGs of GSE19062 and GSE74194 with the MitoCarta3.0 database was done using heatmaps, Venn diagrams, and volcano maps. When multiple transcript IDs were present for a gene, we selected the ID with the highest average expression. Raw gene expression data from all subsequent downstream analyses were log2-transformed and quantitatively normalized. GO/KEGG (http://geneontology.org/docs/go-citation-policy/) enrichment analysis of DEGs was done using the R package ‘clusterProfiler’ [13–15].

### Hub genes and DOMS-infiltrating immune cell analysis

We evaluated the proportion of 22 immune cell types in the skeletal muscle dataset (GSE23697) using the CIBERSORT method and using the R package ‘e1071’. CIBERSORT is a deconvolution algorithm that uses LM22 to infer the proportion of 22 immune cells. LM22 is a reference gene expression signature utilized in the CIBERSORT algorithm to identify and quantify 22 distinct human immune cell subtypes, including various T cell subpopulations, B cells, NK cells, monocytes, and dendritic cells, among others. The reference gene expression signature contains a large number of immune-related genes and can distinguish different immune cell types in diverse samples, thereby aiding the interpretation of the roles of different cell types in disease development. The p-value of each sample deconvolution was obtained, and p < 0.05 was considered accurate. The correlation between each central gene and 22 immune cells was tested by Spearman rank correlation and presented as a lollipop plot.

The ssGSEA (single-sample gene set enrichment analysis) method can be used to determine the relative abundance of different immune cell types in a sample and is widely used for immune cell infiltration analysis in immunology research. In immune infiltration analysis, ssGSEA compares a series of gene sets related to different immune cell types with the gene expression data in a sample to determine the relative abundance of different immune cell types in the sample. Compared with traditional methods based on differential gene expression, ssGSEA can provide more accurate measurement results and can identify very low levels of immune cell types [16]. We used ssGSEA to perform immune infiltration analysis on the GSE23697 dataset. Immune score changes between high and low hub gene expression subgroups were analysed by Wilcox test using the ‘ggpubr’ package (https://github.com/kassambara/ggpubr). Heatmaps and cluster analysis were generated using the ‘ComplexHeatmap’ v2.10.0 package in R to show correlation [17]. Spearman association analysis of immune cells with genes of interest was performed using the ‘pheatmap’ package.

### Evaluation of mitochondrial respiratory chain and mitochondrial metabolism in DOMS by bioinformatics

Oxidative respiratory chain complex DEG calculations were performed on the GSE23697 and MitoCarta3.0 databases using the ‘limma’ package and visualized using the ‘pheatmap’ package. Spearman correlation was used to analyse the correlation between the four hub genes and mitochondrial oxidative respiratory chain complex genes. After that, the results were visualized using the R package ‘ggplot2’ (https://cran.r-project.org/web/packages/ggplot2/ggplot2.pdf).

The Mantel test was used to calculate the correlation between the four hub genes and mitochondrial metabolism, mitochondrial damage (DAMPs), apoptosis, and the correlation coefficient of DOMS and non-DOMS. Visualization was done using the ‘ggcor’ package (https://github.com/houyunhuang/ggcor) [18].

### Statistical analysis

The quantitative results are expressed as means ± standard deviation (SD). Statistical analysis was performed using Student’s t-test or one-way analysis of variance (ANOVA) in GraphPad Prism software. The significance level was set at p < 0.05.

## Results

### Identification and functional enrichment analysis of DEGs related to DOMS skeletal muscle mitochondria

**Fig. 2:**
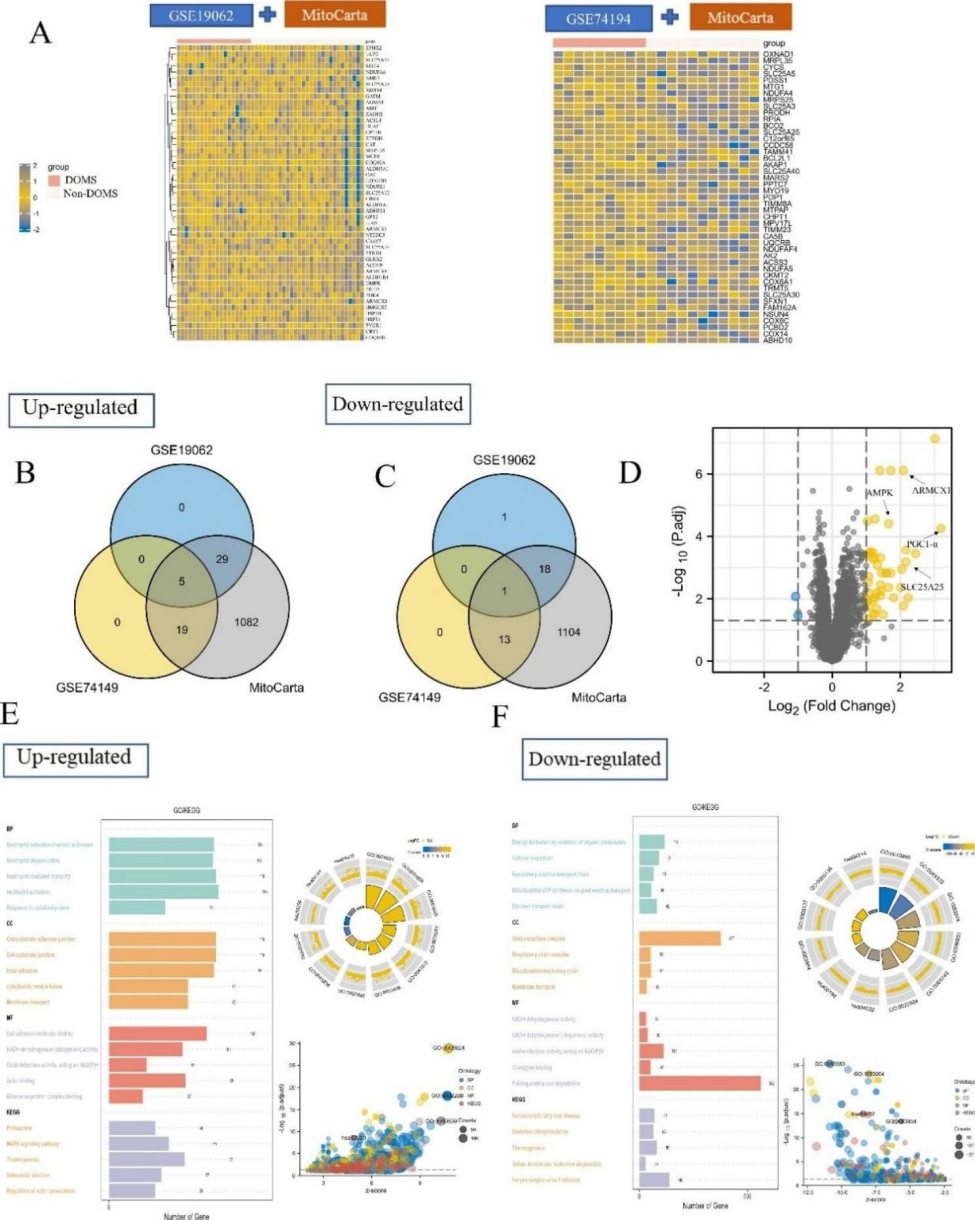
Mitochondrial differently expressed gene enrichment analysis. (**A**) Based on the MitoCarta3.0 database, we extracted mitochondrial differential genes from GSE19062 and GSE74194 public datasets and constructed heat maps. In the heat map, blue represents low expression and yellow represents high expression. We found that the expression of mitochondria-related genes was elevated in DOMS. (**B**, **C**) Display of overlapping mitochondrial differential genes using Venn diagram: (**B**) upregulated genes; (**C**) downregulated genes. (**D**) The volcano map shows the mitochondrial-related differential genes of DOMS and non-DOMS patients. Yellow indicates upregulated genes, blue downregulated genes, and grey non-differential genes. (**E**, **F**) KEGG/GO enrichment analysis of differential genes. The image on the left is the KEGG/GO enrichment analysis, and the bar graph represents the number of differentially expressed genes in each enriched pathway. The image on the right is the enrichment analysis combined with logFC. The logFC of the differentially expressed genes was used to determine whether the corresponding items were positively or negatively regulated: (**E**) upregulated gene enrichment; (**F**) downregulated gene enrichment

In this study, we used GSE19062 and GSE74194 as training cohorts and GSE23697 as a validation cohort. First, we screened for mitochondrial DEGs based on the MitoCarta3.0 database. After that, (log2 | fold-change | > 1) was used as a threshold to screen mitochondrial DEGs. There were 52 mitochondrial DEGs in GSE19062, 48 upregulated and 4 downregulated. There were 45 mitochondrial DEGs in GSE74194, 40 upregulated and 5 downregulated. Visualization analysis of major mitochondrial DEGs was done using a heat map (Fig. 2A). In order to screen hub genes, Venn diagram analysis was performed for upregulated (Fig. 2B) and downregulated genes (Fig. 2 C). Visualization analysis of mitochondrial differential genes was done using volcano maps (Fig. 2D).

We performed a functional analysis of upregulated DEGs. The upregulated KEGG enrichment analysis showed that DOMS is associated with protein processing in the endoplasmic reticulum, the MAPK signalling pathway, and regulation of the actin cytoskeleton. Downregulated KEGG-enriched pathways are involved in oxidative phosphorylation and thermogenesis. In addition, we analysed the DEGs in terms of biological processes, molecular function, and cellular components using the GO database. Upregulated DEGs in DOMS are associated with neutrophil activation involved in immune response, neutrophil degranulation, and neutrophil-mediated immunity. Downregulated DEGs in DOMS are associated with mitochondrial ATP synthesis-coupled electron transport, NADH dehydrogenase activity, oxidoreductase activity, acting on NAD (P) H, quinone or similar compound as acceptor correlation (Fig. 2E, F).

### Identification and validation of mitochondria- and immune-related hub genes

**Fig. 3:**
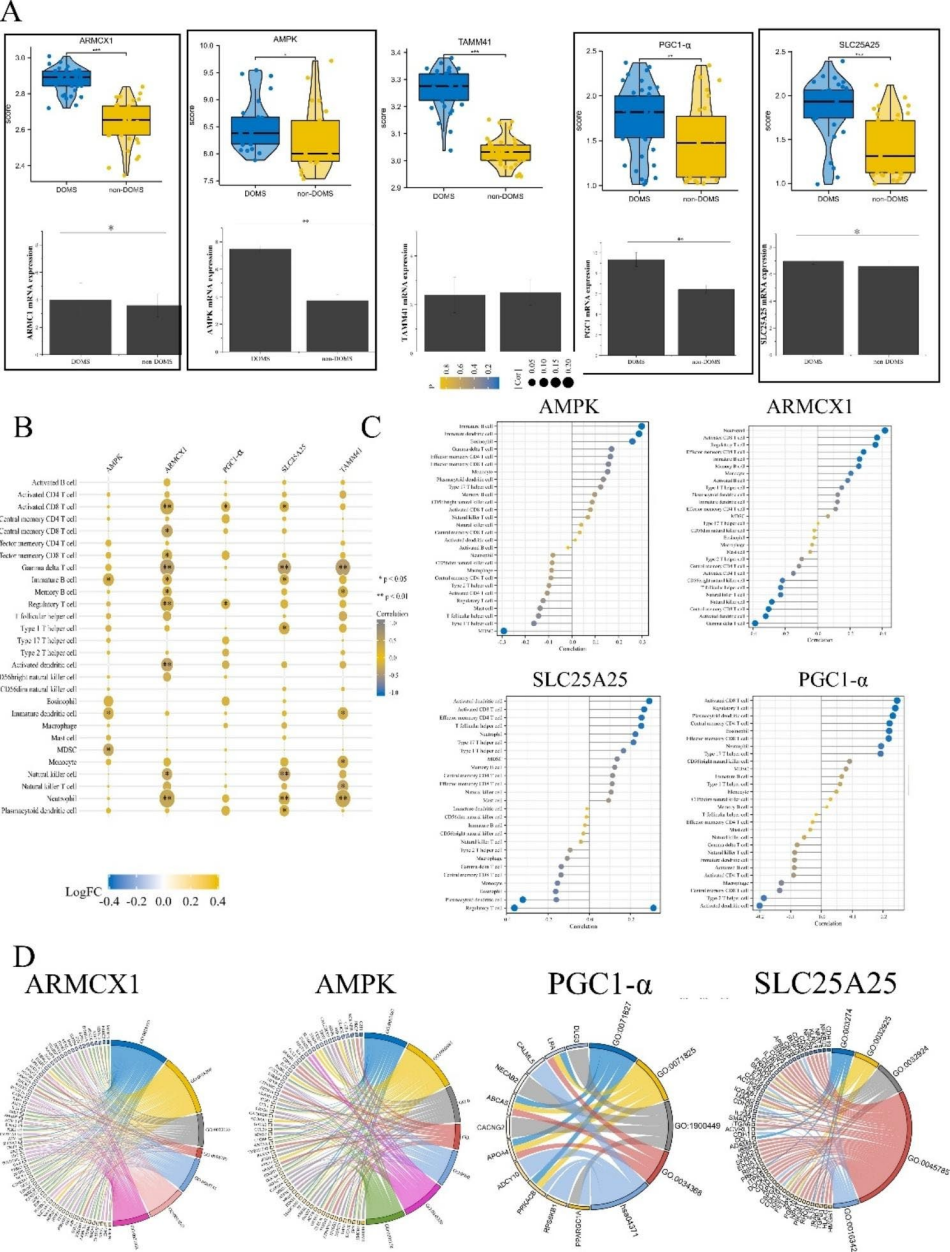
Screening and identification of hub genes. (**A**) Top: five overlapping genes with upregulated mitochondria (*ARMCX1*, *AMPK*, *PGC1-α*, *SLC25A25*, and *TAMM41*). The validation dataset (GSE23697) samples were divided into two groups of non-DOMS and DOMS patients, visualized using the R package ‘ggpubr’; p < 0.05 was set as significant, and one-way ANOVA was used. Bottom: real-time PCR using animal skeletal muscle samples. Data shown were normalized to GAPDH expression and were relative to expression in the non-DOMS (n = 3, error bars represent mean ± SD, *p < 0.05, and **p < 0.01 by Student’s t-test). (**B**) Spearman correlation was used to analyse the correlation between immune cells and hub genes, and a heat map was drawn. (**C**) The size of the lollipop corresponds to the strength of the correlation. (**D**) *ARMCX1*, *AMPK*, *PGC1-α*, and *SLC25A25* correlation gene enrichment analysis

The GSE23697 dataset was used for the validation of differential analysis of *ARMCX1*, *AMPK*, *TAMM41*, *PGC1*, and *SLC25A25*. In addition, real-time PCR was used to verify the hub genes (Fig. 3A). The results showed that *ARMCX1*, *AMPK*, *PGC1-α*, and *SLC25A25* expression was higher in the DOMS group than in the control group. Subsequently, in order to explore the relationship between hub genes and the immune microenvironment, we used the ssGSEA algorithm to calculate the proportion of immune cells in DOMS patients and non-DOMS patients. For example, Fig. 3B, C shows the results of the correlation between the hub genes and immune cells: AMPK was correlated with immature B cells, immature dendritic cells, and eosinophils.

From KEGG enrichment analysis of hub-related genes, the hub genes were found to be associated with autophagy of mitochondria, mitochondrion disassembly, integral components of mitochondrial outer membrane, the MAPK signalling pathway is highly enriched. Interestingly, we found that the four hub genes were highly associated with mitochondrial and immune function (Fig. 3D).

### Relationship between immune cell infiltration, central genes, and the immune microenvironment

**Fig. 4:**
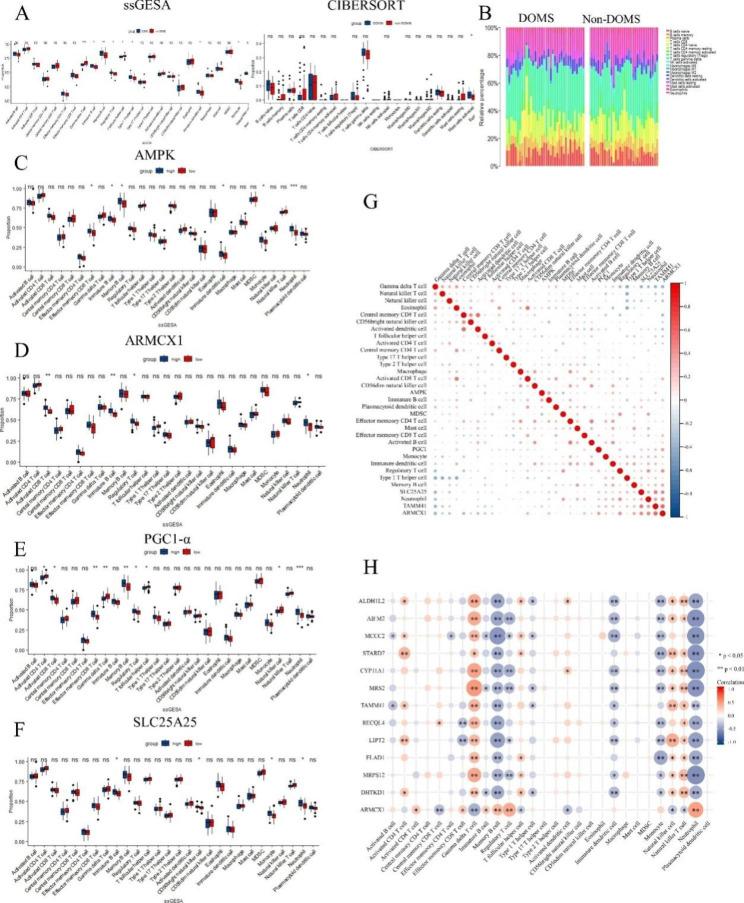
Characteristics of immune infiltration in skeletal muscle of DOMS patients. (**A**) Boxplot of scores based on single-sample gene set enrichment analysis (ssGSEA) in the validation cohort (left); boxplot of CIBERSORT-calculated immune cell proportion scores (right). Red represents non-DOMS patients. Blue represents patients with DOMS. ***p < 0.001, **p < 0.01, *p < 0.05, ns = 1 (independent sample Kruskal–Wallis test). Proportions of immune cells in the DOMS (left) and non-DOMS (right) groups in the validation cohort. (**C**–**F**) Comparison of infiltration by 28 immune cells between the high and low expression groups of four hub genes: (**C**) *AMPK*, (**D**) *ARMCX1*, (**E**) *PGC1-α*, and (**F**) *SLC25A25*. (**G**) Four hub genes (*AMPK*, *ARMCX1*, *PGC1-α*, and *SLC25A25*) were analysed for Spearman correlation with 28 immune cells. (**H**) Analysis of Spearman correlation between mitochondria-related gene (TCA cycle) and 28 immune cells

We calculated the immune cell score of each patient by ssGSEA and CIBERSORT algorithms (data from public datasets). The Kruskal–Wallis test showed that gamma T cells, immature B cell, monocyte, and neutrophil expression in DOMS patients was significantly higher than in non-DOMS patients by ssGSEA (Fig. 4A **left**). The CIBERSORT results showed that the proportion of activated mast cells in DOMS patients was higher than that in non-DOMS ones (Fig. 4A **right**). Figure 4B is a histogram showing the proportion of 22 immune cells in the DOMS and non-DOMS groups. The most common immune cells in the DOMS group were T cells, activated CD4 memory cells (27.5%), activated NK cells (10.8%), plasma cells (18.3%), and M2 macrophages (4.3%). In the samples with high expression of AMPK, ARMCX1, and PGC1-α, the levels of neutrophils, immature B cells, and regulatory T cells were found to be significantly higher compared to the Non-DOMS group (p < 0.05), as shown in Fig. 4 C–E. However, *SLC25A25* shows the opposite trend (Fig. 4F). The correlation heatmap depicted the relationships between 28 distinct immune cell types and hub genes. Notably, AMPK and PGC1-α exhibited predominantly positive correlations with immune cells, suggesting a potential association. Conversely, SLC25A25 and ARMCX1 demonstrated predominantly negative correlations with T cells, while displaying positive correlations with other immune cell populations. These findings underscore the intricate interplay between the hub genes and various immune cell types, indicating their potential involvement in immune regulation and highlighting their relevance in the broader context of immunology research (Fig. 4G).

### Mitochondrial dysfunction in DOMS, including impaired mitochondrial respiratory chain function and impaired mitochondrial metabolism

#### Tricarboxylic acid (TCA) cycle and immune cells

In the current study, we found that the TCA cycle plays a crucial role in regulating immune cells. The possible mechanism is that cellular stress leads to the destruction of the mitochondrial membrane and the release of TCA cycle products into the cytoplasm, resulting in abnormal cellular immunity [19]. In this study, we analysed the correlation between TCA-related molecules and immune cells. Notably, all TCA-associated molecules appear to be negatively correlated with gamma T cells, memory B cells, and neutrophils (Fig. 4H).

### Mitochondrial respiratory chain complex, mtDNA, and immune cells

**Fig. 5:**
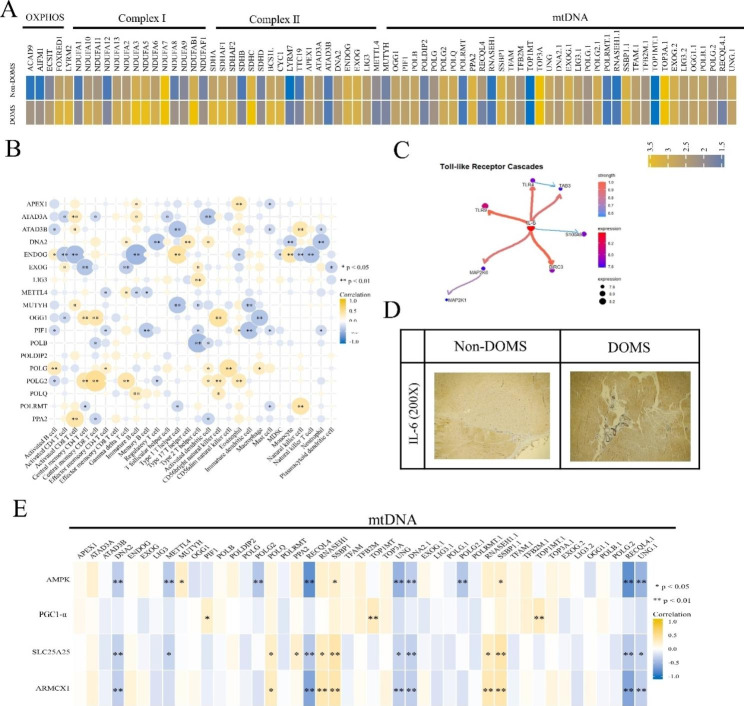
Mitochondrial respiratory chain complex damage and mtDNA expression abnormality in skeletal muscle induced by DOMS. (**A**) Heatmap showing significant upregulation of respiratory chain complexes (I–II) and abnormal mtDNA expression of DOMS in the validation cohort. (**B**) Heatmap of Spearman correlation values between respiratory chain complexes (I–II), mtDNA genes, and immune cells in DOMS from the validation cohort. (**C**) Inference of inflammatory factors and mtDNA using Bayesian neural networks. Scale bar = 50 mm. (**D**) Immunohistochemical staining for IL-6 was performed on paraffin-embedded skeletal muscle specimens from rats. (**E**) Heatmap of Spearman correlation values between mtDNA genes and four hub genes (*AMPK*, *PGC1-α*, *SLC25A25*, and *ARMCX1*) in DOMS from the validation cohort

Mitochondria convert nutrients into ATP mainly through four respiratory chain complexes (I–IV) and ATP synthase (complex V) [20]. Mitochondrial DNA can permeate from mitochondria via the permeability transition pore complex (PTPC). Interferon-β1 (IFNβ1), IL-6, and TNF are then synthesized [21]. In this study, we included the DOMS public data set to systematically understand the effects of DOMS on mitochondrial respiratory chain complexes and mtDNA. The results showed that the expression of mitochondrial respiratory chain complexes and mtDNA was upregulated by DOMS (Fig. 5A). As shown in Fig. 5B, mtDNA (*ENDOG*, *ATAD3B*, *POLB*, and *PIFI*) was negatively correlated with NK cells, T cells, etc. However, *POLG2* and *POLG* were positively correlated with these immune cells. The Spearman correlation analysis showed that the expression of hub genes was negatively correlated with the expression of mtDNA genes *RECQL4*, *UNG*, and *DNA2.1*, and positively correlated with the expression of *SSP1* and *SSP1.1* (Fig. 5E).

A Bayesian network is a causal network, which is considered to be one of the most effective methods to construct gene regulatory networks [22]. In this study, we used the R package ‘CBNplot’ to construct a Bayesian network for inflammatory factors and mtDNA in DOMS. As shown in Fig. 5 C, IL-6 is a major factor regulating inflammatory factors and mtDNA. We identified the localization of IL-6 in DOMS by immunohistochemistry. The results showed that the expression of IL-6 in the non-DOMS group was lower and the distribution was very scattered. However, in the DOMS group, IL-6 expression was evident and concentrated in the damaged area of skeletal muscle (Fig. 5D).

### PGC1-α may be a major protein mediating the mitochondrial and immune microenvironment

**Fig. 6:**
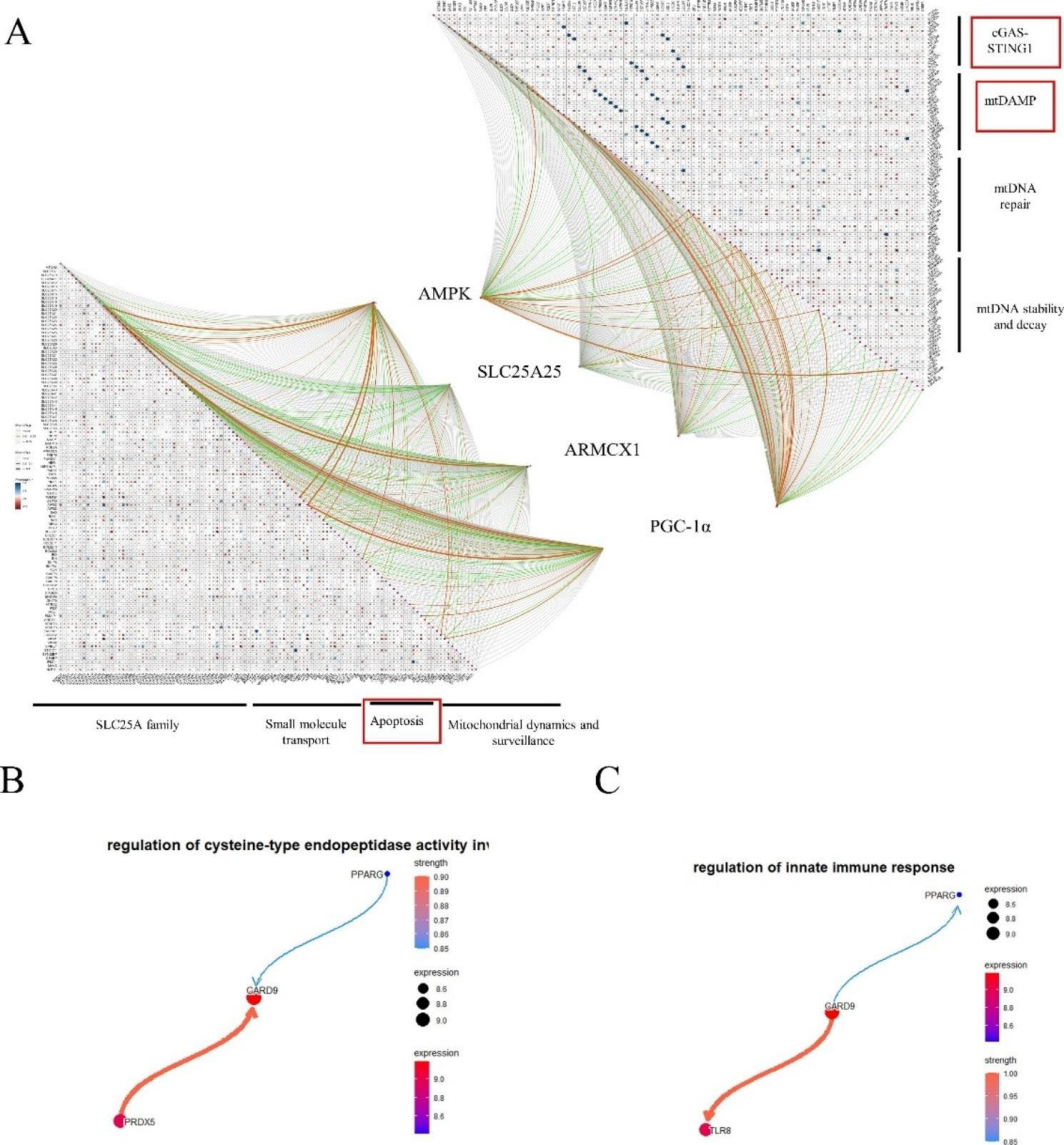
PGC1-α is a major biomarker of mitochondrial dysfunction and the immune microenvironment in DOMS. (**A**). Correlations of four hub genes with mtDNA, apoptosis, and SLC25a family. Colour shows the Pearson’s correlation coefficient between hub genes (*AMPK*, *PGC1-α*, *SLC25A25*, and *ARMCX1*) and mtDNA, apoptosis, and SLC25a family-related genes; blue shows positive correlation (Pearson’s R < 0), red indicates negative correlation (Pearson’s R > 0). The Mantel test was used for statistical analysis; yellow lines indicate a p-value < 0.01, and green lines indicate 0.01 < p < 0.05. *PGC1-α* shows a strong correlation. (**B**, **C**) Construction of Bayesian networks using mitochondrial genes. The related immune pathway showed that *PGC1-α* is the main regulatory gene

**Fig. 7:**
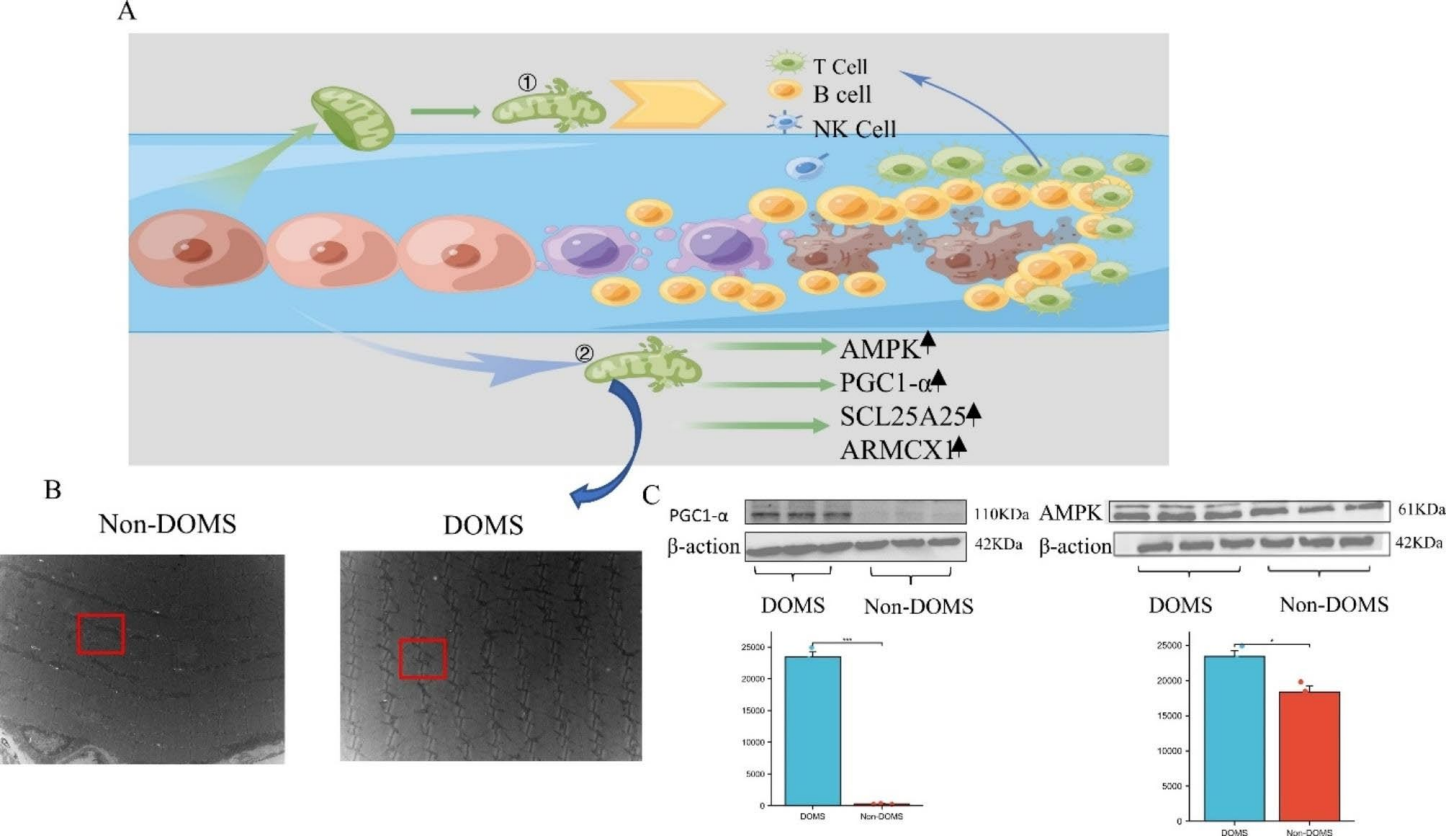
Mitochondria may be a major player in the interaction between inflammation and the immune microenvironment in DOMS. (**A**). Possible mechanism of how mitochondria mediate immune cells in DOMS (①. After intensive exercise, skeletal muscle cells are damaged, and mtDNA in mitochondria is released into inappropriate compartments, acting as PRRs and causing an immune response. ②. Damaged mitochondria produce more oxidative stress. This may further contribute to skeletal muscle damage, including PRR release, massive immune infiltration, and mitochondrial damage). PRRs: pattern recognition receptors. (**B**). Using our own samples for transmission electron microscopy experiments, skeletal muscle mitochondria were swollen and damaged (red arrows) after DOMS. (**C**). Western blot analysis of skeletal muscle samples

Previous studies have found that mtDNA can mediate the expression of immune cells. In addition, mitochondrial damage can also release mtDNA and cause inflammation [21, 23]. In order to explore the relationship between four central genes (*AMPK*, *PGC1-α*, *SLC25A25*, and *ARMCX1*) and mitochondrial metabolism and autophagy, we analysed statistically significant differences using the Mantel test and visualized the results by using the ‘ggcor’ R package. The results showed that the four hub genes (*AMPK*, *SLC25A25*, *ARMCX1*, and *PGC1-α*) were significantly associated with apoptosis, mtDNA maintenance, and mtDNA replication. *PGC1-α* showed a strong correlation (Fig. 6A). Subsequently, we performed Bayesian network analysis on all mitochondrial genes. The results showed that *PGC1-α* is located downstream of multiple pathways (Fig. 6B, C). The mitochondrion is a double-membrane organelle consisting of an inner mitochondrial membrane (IMM) and an outer mitochondrial membrane (OMM). Its dual control layer promotes homologous pattern recognition receptors (PRRs) and mitochondrial DAMP (mtDAMP) [21]. In our study, we believe that intensive exercise leads to mitochondrial membrane damage in skeletal muscle, resulting in abnormal expression of PRRs and mtDAMP, and infiltration of immune cells (Fig. 7A). This idea was subsequently verified in TEM experiments, and we found that the mitochondria of skeletal muscle in rats with DOMS were swollen and the mitochondrial membrane was damaged (Fig. 7B). AMPK and PGC1-α were significantly increased in the Western blot experiment, which was consistent with the bioinformatics results (Fig. 7 C).

## Discussion

DOMS occurs after intense exercise. It causes the muscle strength to drop, leading to muscle ache. However, the DOMS will gradually recover after a period of time. Its pathogenesis has always been a research trend in the field of sports science. The findings of this study provide unique insights into mitochondrial dysfunction and the immune microenvironment in DOMS. We identified and screened four mitochondrial hub genes (*AMPK*, *PGC1-α*, *SLC25A25*, and *ARMCX1*). Recent studies have found that inflammation is usually activated by PRRs on both immune and non-immune cells [24]. Notably, nucleic acids, proteins, and small metabolites do not normally drive the PRR signalling pathway, because they do not go to the PRRs’ subcellular compartments [21]. However, mitochondria can activate the PRR signalling pathway and induce innate and adaptive immunity by regulating cell death [21]. These findings confirm the pathogenicity of inflammation induced by damaged mitochondria. However, the role of mitochondria in DOMS has not been well elucidated. From yeast to humans, AMPK has long been recognized as a major regulatory protein controlling energy balance [25–27]. In addition, the AMPK agonist metformin can stimulate the production of CD8^+^ T cells [28]. In this study, we found that AMPK expression was correlated with T cells and B cells, and AMPK was highly correlated with the mtDAMP signalling pathway. PGC1-α is a transcriptional coactivator that responds to external stimuli. Exercise, caloric restriction, and cold exposure all increase PGC1-α [29]. It has been found that the expression of PGC1-α can increase the number of mitochondria. Overexpression of PGC1-α can increase the content of mtDNA and the proliferation of mitochondria [30, 31]. In addition, it has been found that PGC1-α can enhance CD4^+^ T effector memory cell responses by mediating mitochondrial biogenesis [32]. In our study, PGC1-α was found to be highly correlated with immune-related genes. SLC25A25 is a transport protein. It can enhance Ca^2+^ uptake and induce oxidative stress-mediated cell death [33]. Especially, hub genes were all upregulated in the DOMS group.

The role of immune cells in controlling skeletal muscle repair has been widely researched [34–36]. Earlier studies found that T-cell depletion is associated with skeletal muscle necrosis and fibrosis [37]. In addition, skeletal muscle can cause T cells to accumulate near growth fibres after injury [36, 38]. Subsequent studies have found that T cells regulate skeletal muscle satellite cells mainly through epidermal growth factor and have described the role of T cells in skeletal muscle regeneration, but the results are not detailed [39, 40]. Until now, the composition of the immune infiltrate against DOMS has been expressed mainly by histological staining. Recent advances in next-generation sequencing (NGS), RNA-sequencing (RNA-seq), and computational methods provide unprecedented analysis of transcripts describing immune cell components based on publicly available immune-specific genes. In this study, we applied two algorithms, CIBERSORT and ssGSEA, to deconvolute DOMS immune cell types from public data. Consistent with a previous study, we found an association between immune cell infiltration and DOMS [41]. In this study, we describe the DOMS immune microenvironment in detail. Interestingly, we found that T-cell upregulation was evident after DOMS. After that, we used hub gene expression to group the data set and found that T cells changed significantly in the group with high expression of *AMPK*, *PGC1-α*, and *SLC25A25*.

Inflammatory factors produced after skeletal muscle injury need strong and stable regulation, otherwise skeletal muscle repair will be slowed down [42]. Mitochondria can release mtDNA to activate PRRs and NLRP3, so mitochondria maybe one of the earliest initiators to start up inflammation after muscle injury [43]. IL-6 has dual effects on skeletal muscle injury. On the one hand, IL-6 signals activate early macrophages to invade myoblasts and promote muscle regeneration and myoblast proliferation [43]. Conversely, IL-6 is also a biomarker of adverse outcomes, and skeletal muscle regeneration can be improved by blocking IL-6. In this study, we used Bayesian network inference to find that IL-6 is a major regulatory gene in inflammatory factors. Immunohistochemistry showed that IL-6 is mainly expressed in the injured skeletal muscle. This is consistent with the results of the public validation dataset. Mitochondria have two membranes, the IMM and OMM, which together provide a control layer that separates mitochondrial DAMPs from PRRs [44]. However, once the mitochondrial membrane is damaged, this state will be broken, and DAMPs will activate PRRs and cause the accumulation of immune factors. Interestingly, inflammatory factor-associated genes, along with mitochondria-associated DEGs, were altered with skeletal muscle immune cell infiltration. Another important finding of our study is that mitochondrial respiratory chain complex changes are associated with skeletal muscle immune infiltration.

Mitochondria integrate cell physiology, signalling pathways, and metabolism, thus are important hubs in determining cell fate [45]. In addition, mitochondria can also regulate the metabolism and physiological status of different types of immune cells to coordinate immunity [46]. During the course of DOMS development, immune cells move from metabolic quiescence to activation. Therefore, mitochondrial metabolism and mtDNA have a great impact on immune cell function and activation [47]. Our study found that AMPK, PGC1-α, SLC25A25, ARMCX1 are particularly important in many mitochondrial metabolic pathways. In a previous report, PGC1-α was found to activate CD4^+^ T-cell responses. In our study, PGC1-α may activate immune cells through CARD9 in DOMS. In summary, we found that these hub genes are closely related to gluconeogenesis, the TCA cycle, apoptosis, DAMPs, and other mitochondrial metabolic pathways in DOMS. Damage to mtDNA affects the respiratory chain, enhances oxidative stress, and the inflammatory response, and induces apoptosis. Therefore, mtDNA is considered to be a trigger for stimulating innate immunity, and how mtDNA affects the immune response has become a potential new field in autoimmune diseases.

This study has some limitations. First, the DOMS rat sample is small, and our findings need to be confirmed in a larger cohort in the future. Secondly, although the results of transcriptome-based studies have been validated in rat skeletal muscle, further studies should be extended to clinical applications in the future.

## Conclusion

We identified four hub genes (*AMPK*, *PGC1-α*, *SCL25A25*, and *ARMCX1*) as potential links to the mitochondrial and immune microenvironment. Next, we systematically studied the DOMS immune microenvironment. DOMS is inflammatory pain, which is characterized by inflammatory factors leading to skeletal muscle pain, and mitochondria are considered to be one of the main factors of inflammation. However, the relationship between mitochondria and immune cells in DOMS is still unknown. Our study provides new insights into clinical remission of DOMS.

### Electronic supplementary material

Below is the link to the electronic supplementary material.

Supplementary Material 1

Supplementary Material 2

## Data Availability

The datasets generated and analysed during the current study (GSE19062, GSE74194, GSE23697, GSE23697) are available in the National Center for Biotechnology Information Gene Expression Omnibus (GEO) repository.

## Abbreviations

DOMS: Delayed Onset Muscle Soreness
GEO: Gene Expression Omnibus
DEGs: Differentially Expressed Genes
ROS: Reactive Oxygen Species
TNF-α: Tumor Necrosis Factor Alpha
IL-6: Interleukin-6
qPCR: Quantitative Polymerase Chain Reaction
TEM: Transmission Electron Microscopy
SDS: Sodium Dodecyl Sulfate
PVDF: Polyvinylidene Fluoride
RIPA: Radioimmunoprecipitation Assay
AMPK: AMP-activated Protein Kinase
PGC1-α: Peroxisome Proliferator-activated Receptor Gamma Coactivator 1-alpha
SLC25A25: Solute Carrier Family 25 Member 25
ARMCX1: Armadillo Repeat Containing, X-linked 1
TCA cycle: Tricarboxylic Acid Cycle
DAMPs: Damage-associated Molecular Patterns
LM22: A reference gene expression signature used in the CIBERSORT algorithm
NK cells: Natural Killer cells
RNA-seq: RNA sequencing
GO: Gene Ontology
KEGG: Kyoto Encyclopedia of Genes and Genomes
ssGSEA: Single-sample Gene Set Enrichment Analysis
TCA: Tricarboxylic Acid
mtDNA: Mitochondrial DNA
TEM: Transmission Electron Microscopy
IMM: Inner Mitochondrial Membrane
PRRs: Pattern Recognition Receptors
