# Supplementary figures and images for: A link between mitochondrial damage and the immune microenvironment of delayed onset muscle soreness
Source: BMC Med Genomics. 2023 Aug 23;16:196. doi: 10.1186/s12920-023-01621-9 (PMC10464284; doi:10.1186/s12920-023-01621-9)

## Slide 1
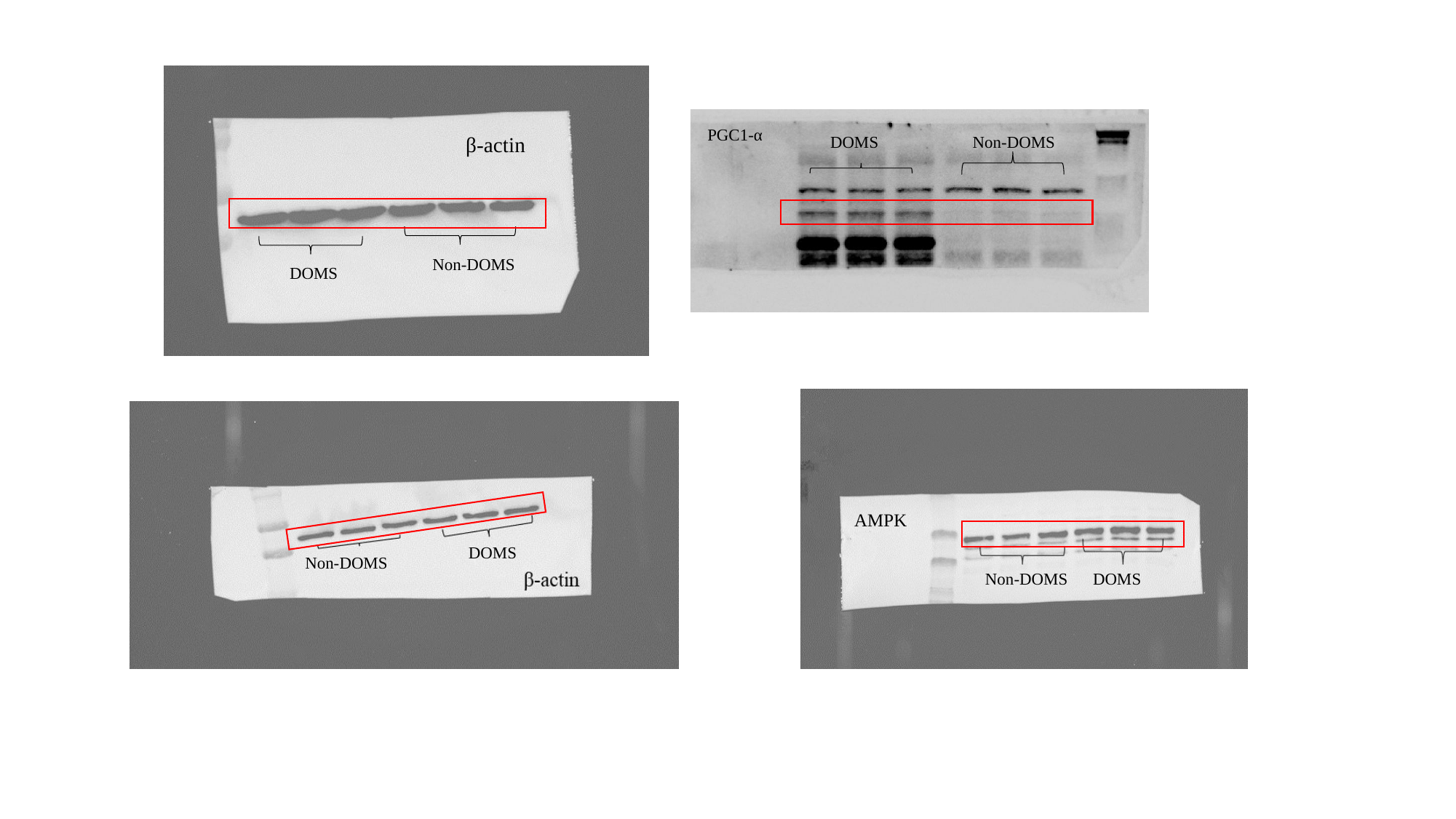

PGC1-α
β-actin
DOMS
Non-DOMS
Non-DOMS
DOMS
AMPK
DOMS
Non-DOMS
Non-DOMS
DOMS

Supplement: Supplementary file 1 — Supplementary Material 1 [file 12920_2023_1621_MOESM1_ESM.pptx]
