# Supplementary material for: A link between mitochondrial damage and the immune microenvironment of delayed onset muscle soreness
Source: BMC Med Genomics. 2023 Aug 23;16:196. doi: 10.1186/s12920-023-01621-9 (PMC10464284; doi:10.1186/s12920-023-01621-9)
Supplement: Supplementary file 2 — Supplementary Material 2 [file 12920_2023_1621_MOESM2_ESM.docx]

***Supplementary Table1***

Li Zheng, Peng Lina, Sun Lili*, Si Juncheng, Zhu Wenning, Yin Weidong

*Corresponding Author: Sun Lili, sunlili@hrbipe.edu.cn

Supplementary Table1: The primer sequences of five biomarkers for qRT-PCR.

| Gene | Sequence（5’-3’） |
| --- | --- |
| *Ampk* | Forward primer TCGGCAAAGTGAAGATTGGA  Reverse primer CTCTTCAACCCTCCCGTGTT |
| *Pgc1-α* | Forward primer TCAGCTGCCTTATTGGTTTCGT  Reverse primer AGCAGCACACTGGTTGGAAG |
| *Slc25a25* | Forward primer CTCTCCTGCCGCCTTCAG  Reverse primer GTGTCTGGAAGAGGCTCCAC |
| *Armcx1*  *TAMM41* | Forward primer GGAGTGTCGGGATGTAGCTG  Reverse primer TCCTTTCCAGCCTCTTGCAG  Forward primer TGCTGGCCTGAAGAAGTCAG  Reverse primer CTCAGCCACCCTTTCCACAT |
| *GAPDH* | Forward primer CGCCAGTAGACTCCACGAC  Reverse primer GCAAGTTCAACGGCACAG |
